# Microstructure and Texture Evolutions During Deep Drawing of Mg–Al–Mn Sheets at Elevated Temperatures

**DOI:** 10.3390/ma13163608

**Published:** 2020-08-14

**Authors:** Jae-Hyung Cho, Sang-Ho Han, Geon Young Lee

**Affiliations:** Korea Institute of Materials Science, 797 Changwondaero, Seongsan-gu, Changwon, GyeongNam 51508, Korea; hsh6188@kims.re.kr (S.-H.H.); arebee@kims.re.kr (G.Y.L.)

**Keywords:** Mg–Al–Mn, magnesium alloys, deep drawing, texture and microstructure, EBSD, dynamic recrystallization

## Abstract

Texture and microstructure evolution of ingot and twin-roll casted Mg–Al–Mn magnesium sheets were examined during deep drawing at elevated temperatures. The twin-roll casted sheets possessed smaller grain sizes and weaker basal intensity levels than the ingot-casted sheets. The strength and elongation at room temperature for the twin-roll casted sheets were greater than those of the ingot-casted sheets. At elevated temperatures, the ingot-casted sheets showed better elongation than the twin-roll casted sheets. Different size and density of precipitates were examined using transmission electron microscopy (TEM) for both ingot-casted and twin-roll-casted sheets. The deep drawing process was also carried out at various working temperatures and deformation rates, 225 °C to 350 °C and 30 mm/min to 50 mm/min, respectively. The middle wall part of cups were mainly tensile deformation, and the lower bent regions of drawn cups were most thinned region. Overall, the ingot-casted sheets revealed better deep drawability than the twin-roll casted sheets. Microstructure and texture evolution of the top, middle and lower parts of drawn cups were investigated using electron backscatter diffraction. Increased deformation rate is important to activate tensile twins both near the bent and flange areas. Ingot casted sheets revealed more tensile twins than twin-roll casted sheets. Increased working temperature is important to activate non-basal slips and produce the DRXed grain structure in the flange. Dynamic recrystallization were frequently found in the top flanges of the cups. Both tensile twins and non-basal slips contributed to occurrence of the dynamic recrystallization in the flange.

## 1. Introduction

Magnesium alloys have limited workability, owing to the limited slip systems at room temperature [1,2,3]. The critical resolved shear stresses (CRSS) of slip and twinning in magnesium alloys strongly depend on the temperature [4,5]. At elevated temperatures, non-basal slip systems can be activated, in addition to basal slips. Accordingly, forming processes can easily be carried out [6].

Non-basal or weak-basal textures are preferred to improve the workability of magnesium sheets. Weakened basal textures can be obtained by adding rare earth metals [7,8]. Instead of a symmetric rolling process, asymmetric rolling is reportedly effective when seeking to alter the microstructures and textures of sheets, i.e., lowering the basal fiber and refining the grain size [9,10,11].

The initial texture and mechanical anisotropy of wrought magnesium sheets strongly affect the drawability [12,13,14,15,16,17]. In addition, the working temperature, head speed, blank holding force (BHF), and amount of lubrication are important for successful drawing [12,18]. Local heating and cooling techniques can improve the formability [19]. The deep drawing of ZK60 alloys has been investigated, and it was found that twin-roll casted (TRC) sheets had better deep-drawability than ingot-casted (IC) sheets [20]. Dynamic recrystallization (DRX) and peak splitting along the rolling direction (RD) were mainly observed in the TRC sheets, which implied that these factors contribute to better drawability.

The Mg–Al–Mn alloying system possesses AlMn dispersions. This results in a smaller grain size than pure magnesium alloys [21]. In the Mg–3Al–0.5Mn–0.2Mm system, TRC sheets show better yield and tensile strength levels than IC sheets [22]. Using differential speed rolling in the AM31 system, improved strength, and ductility were realized through grain refinement and a weakened basal texture [23]. DRX behavior during the uniaxial compression of the AM30 system was also investigated in detail [24]. At a low strain rate of $0.1\;s^{-1}$, effect of twinning on the microstructure and textural evolution was negligible. At a greater strain rate of $0.5\;s^{-1}$, a high volume fraction of twins was activated, but they were consumed by DRX with strain. Dominant deformation modes varied considerably, depending on the loading direction along the extrusion direction or radial direction. The effect of fine second-phase particles such as Al-Mn intermetallics on microstructural evolution during annealing was also found in Al alloys [25,26]. The second-phase particles had a strong effect on microstructural evolution during the hot deformation and recrystallization of the as-cast Al structures.

In this study, flow behaviors under uniaxial tension of Mg-Al-Mn sheets fabricated by TRC and IC processes were examined at room and elevated temperatures. Deep drawing was also carried out on two different Mg-Al-Mn sheets under various working temperatures and punch speeds. Using EBSD (electron backscatter diffraction) and TEM (transmission electron microscopy), the microstructure, texture and second phases were characterized and examined to gain a better understanding of deformation behaviors of the Mg-Al-Mn sheets. In particular, the characteristics of textural and microstructural evolution during deep drawing were investigated based on EBSD in detail. The variation of deformation modes was discussed according to different deformation history during drawing.

## 2. Materials and Methods

### 2.1. Materials

The chemical compositions of the magnesium alloys used here were Mg—3.3 Al—0.78 Mn (AM31) in terms of the weight. A twin-roll caster with a roll diameter of 300 mm was used to fabricate the strips. Detailed information about TRC process has been discussed in reference [27]. Molten alloy with an initial temperature of 750 °C was used for the TRC process. The rolling speed during the TRC process was 3 rpm (revolution per minute). The strips were approximately 4.0 mm thick and 180 mm wide. In order to fabricate the IC strips, molten alloy at 720 °C was poured into a steel mold 180 mm long, 160 mm wide, and 25 mm thick. The IC slab was heat-treated at 400 °C for 20 hrs for homogenization, after which it was cut to a thickness of 4 mm for a comparison with the TRC strips.

To fabricate the sheets for deep drawing, approximately seven passes of warm rolling were carried out on the slabs and strips with a thickness of 4 mm. The thickness of the workpieces was reduced from 4 mm to 0.6 mm. Working rolls with a diameter of 200 mm were preheated to 250 °C and operated at a speed of 3.5 rpm. Before warm rolling, the workpieces were heated at 350 °C for 30 min. The intermediate annealing time and temperature between the passes were 5 min and 350 °C, respectively. Final annealing was carried out on the sheets with a thickness of 0.6 mm for 1 hr at 350 °C.

### 2.2. Deep Drawing

Deep drawing was carried out using a universal sheet testing machine (USTM, YoungJin Precision, Korea). The punch diameter was 37 mm and the inner diameter of the die was 39 mm. The diameter of the flange was 74 mm and limit drawing ratio was 2.0. The punch speeds during drawing step were 30, 40, and 50 mm/m, and the working temperatures ranged from 200 °C to 350 °C. The die was heated to a specific temperature and the workpieces were laid on it. The workpieces were then held about 5 min to reach the temperature of the die. BHF during drawing was about 5∼12 kN, and it varied with deep drawing conditions, as summarized in Table 1. Detailed information on deep drawing has also been presented in reference [20]

### 2.3. Microstructural Characterization and Mechanical Testing

The microstructure and second phases were characterized using SEM (scanning electron microscopy) and TEM (transmission electron microscopy). Microstructure and microtexture analyses were carried out using an automated high-resolution EBSD (HKL Channel5, Oxford, UK) attached to the SEM device (JEOL7001F, Jeol, Tokyo, Japan). The EBSD samples were mechanically polished and then electropolished using a solution of butyl cellosolve (50 mL), ethanol (10 mL), and perchloric acid (5 mL) at a voltage of 10 V and temperature of −15 °C to −20 °C. The step size for EBSD mapping was 0.5 μm. The precipitates were examined using TEM (JEM-2100F, Jeol, Tokyo, Japan) operating at 200 kV. The TEM samples were prepared by mechanical polishing down to a thickness of approximately 100 μm, followed by electropolishing using methanol (60 mL), glycerin (30 mL), and nitric acid (10 mL). Electropolishing was carried out at a temperature and voltage of −10 °C to −15 °C and 20 V to 25 V, respectively. These samples were finalized by ion thinning to prevent oxidation or other types of surface contamination. A quantitative analysis was carried out using energy dispersive spectroscopy (EDS).

Uniaxial tension tests (ASTM E8 standard) were carried out along the rolling direction (RD) using a standard universal testing machine (Instron 4206, Norwood, MA, USA). The gauge length and width of the samples were 12.5 mm and 3 mm. The strain rates were 0.001/s and 0.002/s at various temperatures.

## 3. Results

### 3.1. Initial Microstructure

**Figure 1:**
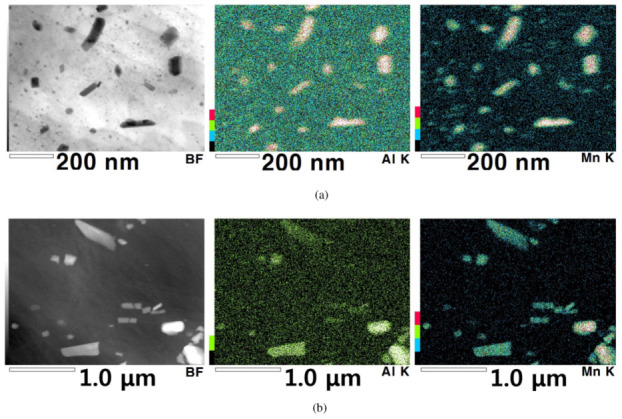
Micrographs of various second phases and their energy dispersive spectroscopy (EDS) results. (**a**) TRC, and (**b**) IC.

Various second phases and their EDS results for Mg–Al–Mn sheets are presented in Figure 1. The size and density of the second particles depend on the IC and TRC fabrication processes. The IC samples contain larger particles than the TRC samples. Instead, numerous refined particles are observed in the TRC samples. The second particles in the Mg–Al–Mn sheets mainly consist of Al and Mn, implying ${\mathrm{Al}}_{8}{\mathrm{Mn}}_{5}$ dispersions [28].

**Figure 2:**
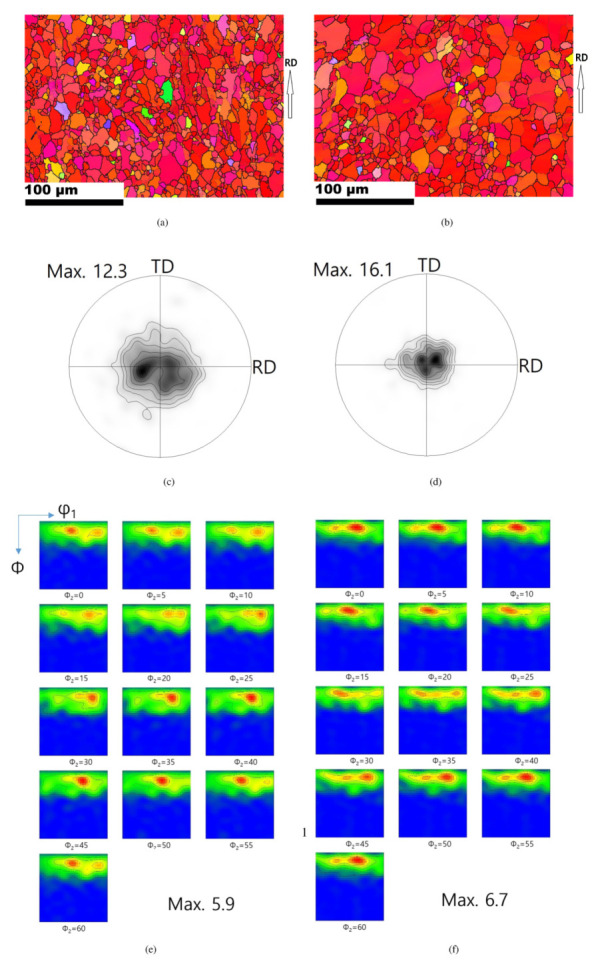
Inverse pole figures (IPFs) maps, pole figures (PFs) and orientation distribution functions (ODFs) of the initial sheets. PF contours: 1, 2, 3, 5, 7, 10. ODF contours: 1, 2, 3, 4, 5, 6. IPFs for (**a**) TRC, and (**b**) IC. 0001 PFs for (**c**) TRC, and (**d**) IC. ODFs for (**e**) TRC, and (**f**) IC.

Inverse pole figure maps (IPFs), pole figures (PFs), and orientation distribution function (ODF) of the initial Mg-Al-Mn sheets are shown in Figure 2. Grain identification angles (GID) of 2° and 15° were used for the low and high angle grain boundaries, respectively. The TRC and IC sheets both mainly possessed strong basal orientations. The overall grain sizes of the TRC sheets (16 μm) are smaller than those of the IC sheets (25 μm), as shown in Figure 2. Detailed crystallographic information can be better understood from the PFs. The rolling direction (RD), transverse direction (TD), and normal direction (ND) are located in the right, top, and center of the 0001 PF. The peak intensity of the TRC sheets is lower than that of the IC sheets. Both the TRC and IC sheets showed a split of the basal peak along the rolling direction (RD). A pyramid 〈c+a〉 slip can split the basal intensity toward the RD and weaken the basal texture during hot rolling [1]. At elevated temperatures, the CRSS of non-basal slips usually decreases. Double twinning of $<10\bar{1}1>-<10\bar{1}2>$, with $<10\bar{1}1>$ compressive twinning and followed by $<10\bar{1}2>$ tensile twinning also provides a peak split along the RD [29,30]. Double twinning mainly possesses misorientation angles of 37.5° or 30.1° about the common zone axis of $<11\bar{2}0>$ for magnesium [31,32]. ODFs of initial sheets are also presented. When considering rolled magnesium sheets, hexagonal crystal and orthorhombic sample symmetries are assumed in Euler space, i.e., {0–90°,0–90°,0–60°}. Strong peaks are located along ϕ=15°–20° in ODF sections, and this implies deviation of basal fiber from the ideal position (ϕ=0°) due to split.

### 3.2. Mechanical Behaviors

**Figure 3:**
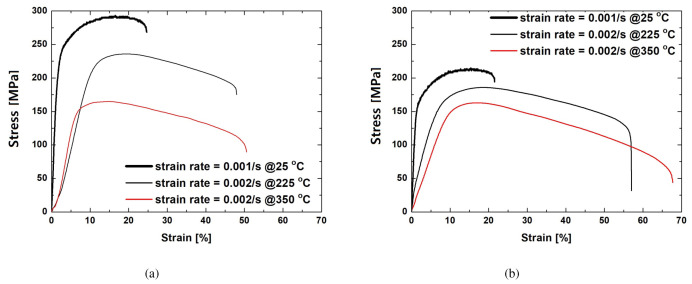
Tensile stress-strain curves of Mg–Al–Mn sheets at various temperatures and strain rates. (**a**) TRC, and (**b**) IC.

**Figure 4:**
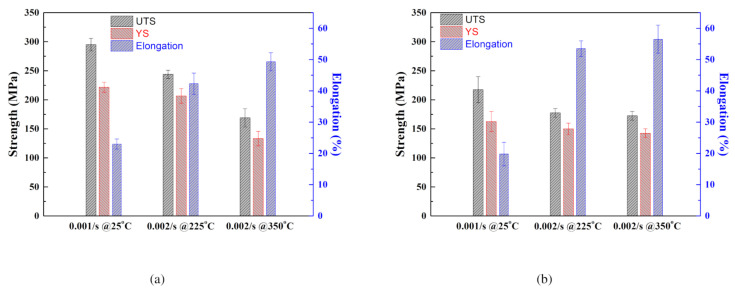
Mechanical properties of Mg-Al-Mn sheets. UTS: ultimate tensile strength [MPa]. YS: yield strength [MPa]. (**a**) TRC, and (**b**) IC.

The typical flow curves of the Mg–Al–Mn sheets used are presented in Figure 3. The TRC samples show greater strength and elongation than the IC samples at room temperature. The tensile strengths of the TRC and IC samples are approximately 290 MPa and 210 MPa, respectively, and their corresponding elongations are 25% and 22%. It has been reported that TRC samples possess both greater strength and elongation than IC samples in ZK60 alloys [20] at room temperature. In Mg–Al–Mn alloys with Ca or misch metals, however, strength of TRC samples is greater than that of IC, and elongation of TRC is smaller than that of IC [22,28]. They seem to comply with trade-off relationship between strength and elongation. At elevated temperatures of 225 °C and 350 °C, this studies on Mg–Al–Mn are similarly reflecting the previous results of Mg-Al-Mn alloys with Ca and misch metals. The elongations of the IC samples are greater than those of TRC samples. Young’s modulus of metals usually decreases with increase in temperature, although it has some variations due to second-phase particles and defects [33,34]. Overall, these trends are also found in Mg–Al–Mn alloys. Figure 4 summarized mechanical properties of Mg-Al-Mn sheets. Increased elongation of IC sheets at elevated temperatures is noticeable.

### 3.3. Deep Drawing Process

**Figure 5:**
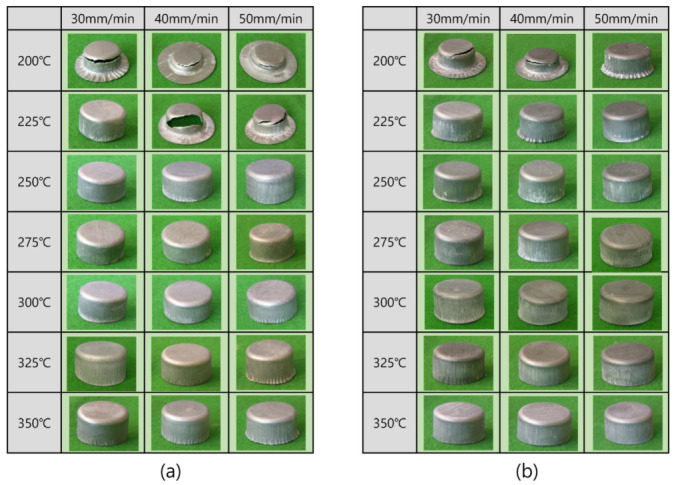
Drawn cups made of Mg–Al–Mn sheets at various temperatures and deformation rates. (**a**) TRC, (**b**) IC.

**Figure 6:**
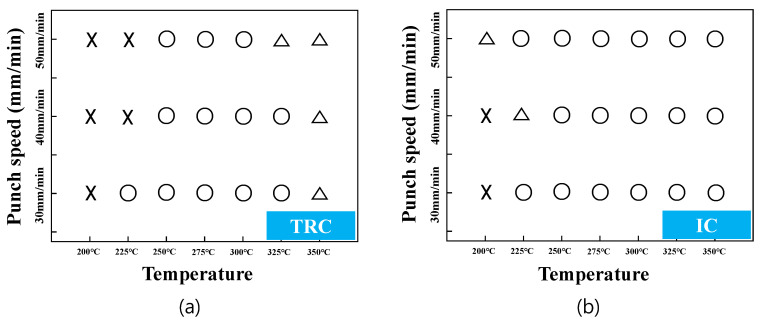
Deep drawability of Mg–Al–Mn sheets at various temperatures and deformation rates. ×: failed, ◯: succeeded, ∆: wrinkled. (**a**) TRC, (**b**) IC.

Photos of the drawn cups made of Mg-Al-Mn sheets are shown in Figure 5. Working windows of deep-drawing are presented according to various temperatures and deformation rates, as shown in Figure 6. At 200 °C, both TRC and IC sheets failed during deep drawing. At 225 °C, the IC sheets revealed better drawability than TRC sheets with cracks. At temperatures greater than 225 °C, the deep drawing process was successful with the IC sheets. It appears that the improved elongation of IC samples at elevated temperatures resulted in better workability when considering the flow behaviors as shown in Figure 3. There were some wrinkles in the top flange of the TRC cups, even at 350 °C. Those wrinkles in the top flange is related to module design (friction or blank-holder force) and materials’ mechanical properties (such as anisotropy and work hardening and microstructural inhomogeneity). Although it was possible to reduce the wrinkles in the top flange with more delicate module design, the TRC sheets resulted in minor wrinkles in the top flange during deep drawing. Note that no wrinkles were observed in the flange of the IC sheets. This seems to be associated with different mechanical responses of the TRC sheets from those of IC sheets at the temperature. More detailed microstructural features of drawn cups will be discussed later.

**Figure 7:**
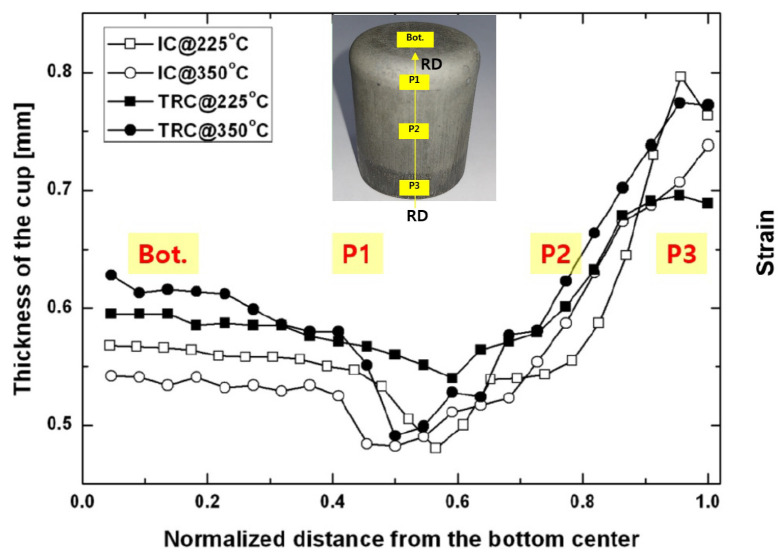
Variations of thickness during deep drawing.

Figure 7 shows the variations of the thickness during the deep drawing of the Mg-Al-Mn sheets. From the bottom to the top flange, the thickness variations of the TRC and IC sheets appear to be similar to each other. The bottom (Bot.), Part 1 (P1), Part 2 (P2), and Part 3 (P3) regions are aligned with the rolling direction of the sheets. The EBSD measurements were carried out from each position, as specified in the small inset. Each region reflects different deformation history during deep drawing process. Part 1 is the bent region corresponding to the punch radius. Part 2 is the middle region of the drawn cup. Tensile loading was mostly applied to the cup wall and, thus, thinning of the wall could be clearly observed. The narrowest thickness of the wall was also found between Part 1 and Part 2. Most instances of failure or necking occur typically in this region. Past Part 2, the wall thickness increased when compared to the initial thickness, as the compressive metal flow along the circumferential direction thickens the flange part. Occasionally, this part experiences ironing between the tool and the punch due to the increased thickness. In this experiment, large clearance was allowed to avoid the ironing effect. Part 3 corresponds to the top flange, where most of the thickening was observed due to the severe compressive loading along the circumferential direction. This results in tensile twinning in the Mg sheets. Microstructure evolution during deep drawing will be discussed later in more detail.

### 3.4. Microstructure Evolution During Deep Drawing

**Figure 8:**
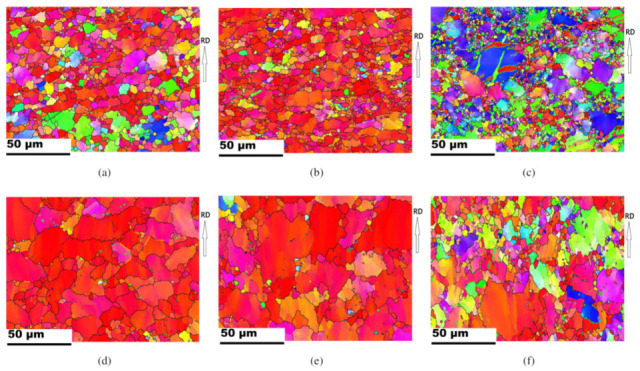
IPF maps of Part 1, Part 2, and Part 3 regions of drawn cups. The punch speed and working temperature during deep drawing were 30 mm/min and 225 °C, respectively. Each twin boundary of tensile, compressive, and double twins is specified with green, blue, and yellow. (**a**) Part 1, (**b**) Part 2, and (**c**) Part 3 for TRC sheets. (**d**) Part 1, (**e**) Part 2, and (**f**) Part 3 for IC sheets.

Figure 8 presents inverse pole figure maps of Part 1, Part 2, and Part 3 of drawn cups processed at a punch head speed of 30 mm/min and at a temperature of 225 °C. The evolution of the microstructure and texture during the deep drawing process was investigated by means of EBSD. Grain identification angles of 2° and 15° were used for the low and high angle grain boundaries, respectively. Three different twin boundaries are specified with contrasting colors. Tensile twin boundary ($86{}^{\circ}<11\bar{2}0>$) is specified in green, compressive twin boundary ($56{}^{\circ}<11\bar{2}0>$), blue, and double twin boundary ($38{}^{\circ}<11\bar{2}0>$), yellow. The initial grain size of the TRC sheet was smaller than that of the IC sheet, and the microstructure of the drawn cup reflected this. As discussed earlier, each part reflected the corresponding deformation history during deep drawing. For a comparison of only the crystallographic reorientation without rigid body rotation caused by the working dies, a sample coordinate system is made to adhere to the sheet. Part 1 experienced bending due to curvature of the punch radius. The actual strain was not significant in the Part 1 and minor textural evolution was usually observed. Figure 8a illustrates rather off-basal orientations, which implies some activation of non-basal slips during bending of the TRC sheets. Part 2 mainly experienced tensile deformation, and this resulted in slip deformation rather than twinning. Part 3 near the flange showed drastic reorientation due to the severe compressive loading along the circumferential direction. Both large grains with tensile twin boundaries and refined small grains were observed due to severe deformation in Part 3, as shown in Figure 8c. In particular, equi-axed small grains were found along with deformed large grains in the TRC sheet, implying DRX. The second particles in Mg alloys enhanced the activity of non-basal slips and promoted the DRX [35]. Strong activation of tensile twins also contributes to DRX in Mg–Al–Mn (AM30) alloys [24]. Both non-basal slips and tensile twins can cause DRX in the flange during cup drawing. When compared to the TRC sheet case, the IC outcome evidently revealed remained initial orientations of basal fibers during bending, as shown in Figure 8d. Small grains were also found along with large grains in Part 3 of the IC sheet. The volume of small grains in Part 3 of the IC sheet was limited, as compared to that of TRC sheet.

**Figure 9:**
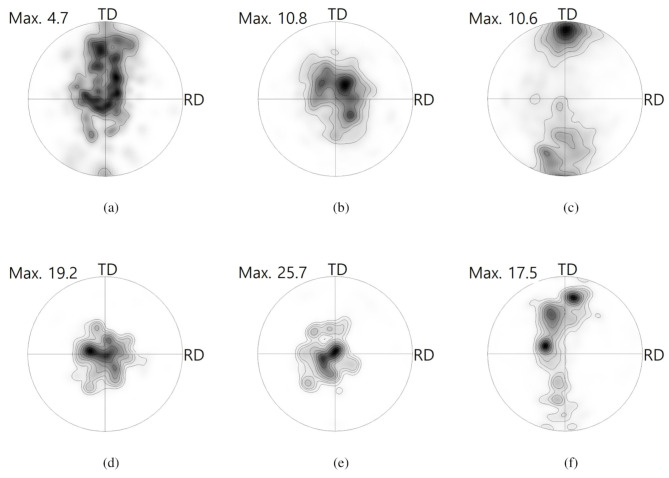
0001 PFs computed from EBSD maps, as shown in Figure 8. Contours: 1, 2, 3, 5, 7, 10, 15, 20. (**a**) Part 1, (**b**) Part 2, and (**c**) Part 3 for TRC sheets. (**d**) Part 1, (**e**) Part 2, and (**f**) Part 3 for IC sheets.

More detailed texture evolution is better understood in PFs, as shown in Figure 9. In the TRC case, the PF of Part 1 has some peak intensities along the ND to the TD. Peak split along the TD reflects activation of prismatic 〈a〉 slips [29,36,37]. Part 2 revealed a wide basal texture, which deviated from the ND. In the flange of Part 3, strong peak intensity around the TD implies active occurrence of tensile twinning, as shown in Figure 9c. The thickening of the sheets can contribute to the activation of tensile twinning of Mg sheets. Crystallographically, tensile twinning can be activated under tensile loading throughout the thickness of Mg-Al-Mn sheets with strong basal textures. Basal intensity rarely remained in the center of the 0001 PF of Part 3. Most of refined grains in the TRC sheets implied DRX after tensile twinning. In the IC case, basal fibers remained in Part 1 and Part 2. In Part 3, reorientation that was caused by non-basal slips and minor tensile twinning seemed to occur. Non-basal slip of prismatic 〈a〉 is more likely responsible for peak spreading along the TD. Refined DRX in the IC sheet seemed to be associated with the non-balsa slips, rather than tensile twins. Note that major deformation modes differ in the bent and flange of the TRC and IC sheets.

**Figure 10:**
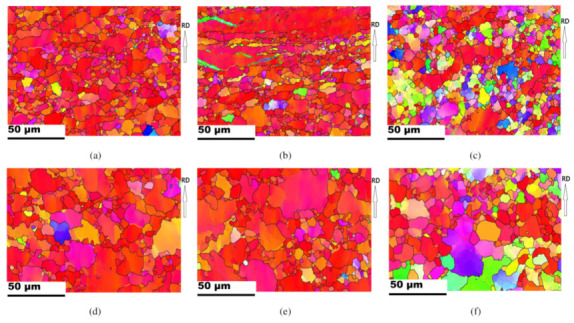
IPF maps of Part1, Part 2 and Part3 regions of drawn cups. The punch speed and working temperature were 30 mm/min and 350 °C, respectively. (**a**) Part 1, (**b**) Part 2, and (**c**) Part 3 for TRC sheets. (**d**) Part 1, (**e**) Part 2, and (**f**) Part 3 for IC sheets.

The texture and microstructure evolution during deep drawing at a punch speed of 30 mm/min at a temperature of 350 °C are shown in Figure 10. Overall, the microstructural features are similar to the earlier outcomes, as shown in Figure 8, except Part 3, where the most severe deformation (thickening) occurred. It should be noted that tensile twinning was reduced in Part 3 for both TRC and IC as compared to that at low temperature of 225 °C. The punch speeds are identical in both cases and thus the microstructural difference between them appears to come from the increased working temperature. The increase in the working temperature can affect the CRSS of the deformation modes. This implies that non-basal slip systems to accommodate external compressive loading along the circumferential direction are activated instead of twinning.

**Figure 11:**
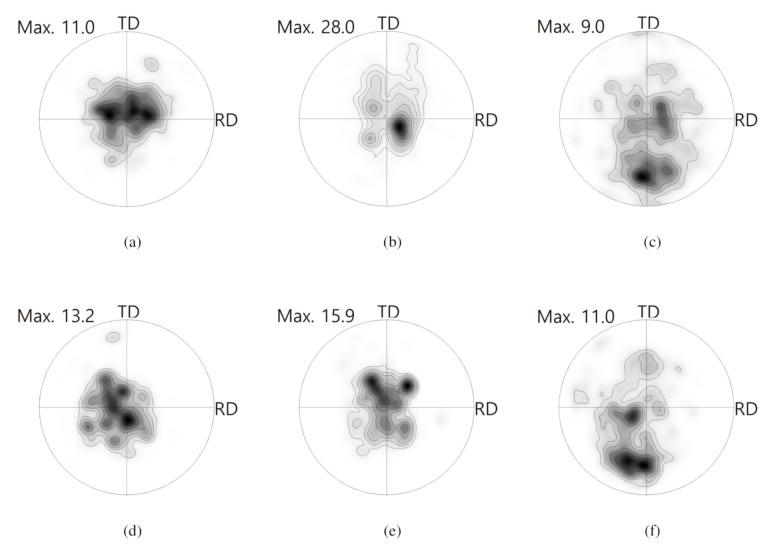
0001 PFs computed from EBSD maps, as shown in Figure 10. Contours: 1, 2, 3, 5, 7, 10, 15, 20. (**a**) Part 1, (**b**) Part 2, and (**c**) Part 3 for TRC sheets. (**d**) Part 1, (**e**) Part 2, and (**f**) Part 3 for IC sheets.

Based on the PFs computed from the EBSD data in Figure 10, as shown in Figure 11, it was found that the crystallographic reorientation during drawing at 350 °C did not differ greatly from that at 225 °C, except Part 3. It is evident that less twinning occurred in Part 3 at 350 °C when compared to that at 225 °C. Based on the slip system activity, splitting of the basal intensity along the TD is associated with the activation of the prismatic <a> slip system. At an increased temperature, prismatic <a> slip can be more easily activated and result in the formation of a peak split [5,38].

**Figure 12:**
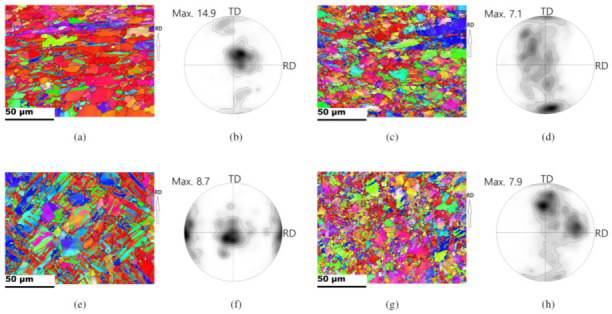
IPF maps and computed 0001 PFs of Part1, and Part3 regions of drawn cups. The punch speed and working temperature were 50 mm/min and 225 °C, respectively. Contours: 1, 2, 3, 5, 7, 10, 15, 20. TRC sheets: (**a**) IPF, and (**b**) 0001 PF for Part 1; (**c**) IPF, and (**d**) 0001 PF for Part 3. IC sheets: (**e**) IPF, and (**f**) 0001 PF for Part 1; (**g**) IPF, and (**h**) 0001 PF for Part 3.

When increasing the deformation rate, the microstructural evolution can be affected. Figure 12 presents the microstructure and pole figures at an increased deformation rate of 50 mm/min at 225 °C. Some tensile twin boundaries are found in Part 1 of the TRC sample (Figure 12a). Most evident tensile twin boundaries are observed in the IC sample, as shown in Figure 12e. Those tensile twin boundaries are rarely found at a deformation rate of 30 mm/min (Figure 8a). In part 3, at the increased deformation rate of 50 mm/min, IPFs of both the TRC and the IC sheets provide a strong evidence of DRX. Figure 12c,g reveal that refined grain structures arose in both the TRC and IC sheets due to DRX. Deformed large grains are also observed along with DRX grains. It is likely that the increased deformation rate from 30 mm/min to 50 mm/min mainly contributes to the increases in the tensile twinning and followed by DRX.

0001 PFs computed from the EBSD maps are also presented. Peaks near the TD imply tensile twinning activation, and Figure 12b illustrates minor twinning in Part 1 of the TRC sheet. In fact, deep drawing of the TRC sheet at a deformation rate of 50 mm/min at 225 °C was unsuccessful, and there was a crack near Part 1 (bent region). Drawing of the IC sheet under the same condition of Part 1 was successful. Strong peaks that are near the RD caused by tensile twinning are confirmed in the IC sheet, as shown in Figure 12f. At a high deformation rate, twinning activation can play an important role in accommodating external loading. It is feasible that limiting the active deformation modes will result in a failure in the TRC sheet. The activation of tensile twins in the IC sheet is dominant, compared to that of the TRC sheet. It surely supports that larger grain size is favorable for twinning activation. It appears that active tensile twins improved the bendability near the bent region. In Part 3, strong off-basal textures were observed in both TRC and IC sheets, which originated from tensile twinning and non-basal slips.

**Figure 13:**
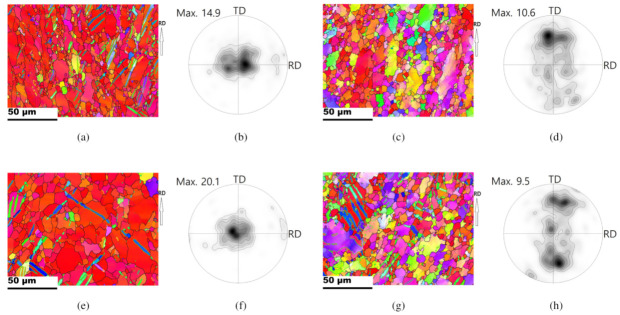
IPF maps and computed 0001 PFs of Part1, and Part3 regions of drawn cups. The punch speed and working temperature were 50 mm/min and 350 °C, respectively. Contours: 1, 2, 3, 5, 7, 10, 15, 20. TRC sheets: (**a**) Part 1, and (**b**) Part 3 for IPFs; (**c**) Part 1, and (**d**) Part 3 for PFs. IC sheets: (**e**) Part 1, and (**f**) Part 3 for IPFs; (**g**) Part 1, and (**h**) Part 3 for PFs.

At the deformation rate of 50 mm/min, the microstructure at a temperature of 350 °C is different from that at 225 °C (Figure 13). Tensile twins in Part 1 are relatively reduced at 350 °C when compared to those at 225 °C. DRX in Part 3 also varies with working temperature. The equi-axed grains at 350 °C are much larger than those at 225 °C. The equi-axed grain structures at 350 °C at the deformation rate of 50 mm/min (Figure 13c,g) have the similarity to those at 350 °C at the deformation rate of 30 mm/min (Figure 10c,f). It seems that equi-axed grains correspond to the region that was caused by DRX, followed by grain growth, which is more substantial at 350 °C than at 225 °C due to the increased working temperature. Deformed regions mainly correspond to the large initial grains, inside which low angle misorientations and tensile twins are observed in the TRC and IC samples, respectively. Increasing the temperature from 225 °C to 350 °C caused a change in the deformation mechanism in the flange, and the twinning activation was reduced. The 0001 PFs also reflected this change in the crystallographic reorientations of off-basal textures.

## 4. Discussion

**Figure 14:**
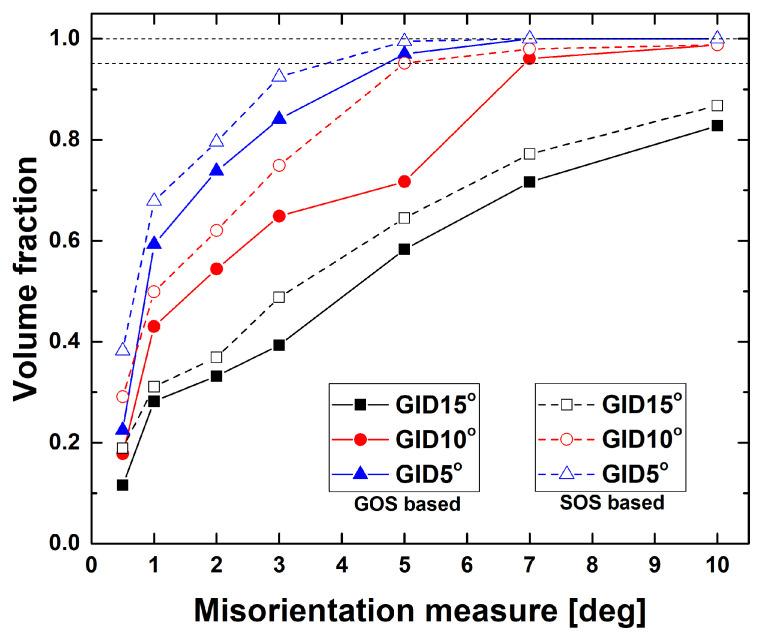
Misorientation measures of GOS and SOS obtained from initial microstructure of Mg-Al-Mn sheets.

The deformation of Mg alloys at elevated temperatures frequently results in DRX. The crystallographic orientations of newly recrystallized grains are similar to those of the deformed grain structure, which implies continuous DRX [39,40]. Static recrystallization of Mg alloys also revealed little change of crystallographic texture, although recrystallization near twinning or shear band contributed to texture randomization or weakening [41,42]. In this study, Mg–Al–Mn alloys also disclosed DRX during deformation at elevated temperatures. Based on initial microstructure of Mg–Al–Mn sheets, two different misorientation measures were computed in Figure 14. Grain orientation spread (GOS) and scalar orientation spread (SOS) can be used as measures to differentiate deformed and recrystallized grains. Each measure expresses the magnitude of misorientation among all pixels in a grain and that of between each pixel and the average orientation, respectively [43]. Misorientation measure depends on grain identification angles (GID). Figure 14 represents the volume fraction of deformed and recrystallized grains, based on each criterion of GOS and SOS. Initial microstructure can be assumed to be recrystallized and, thus, the criterion to collect more than approximately 95% of recrystallized grains can be used. The criterion of SOS misorientation measure of 5° and GID angle of 10° looks reasonable for the purpose.

**Figure 15:**
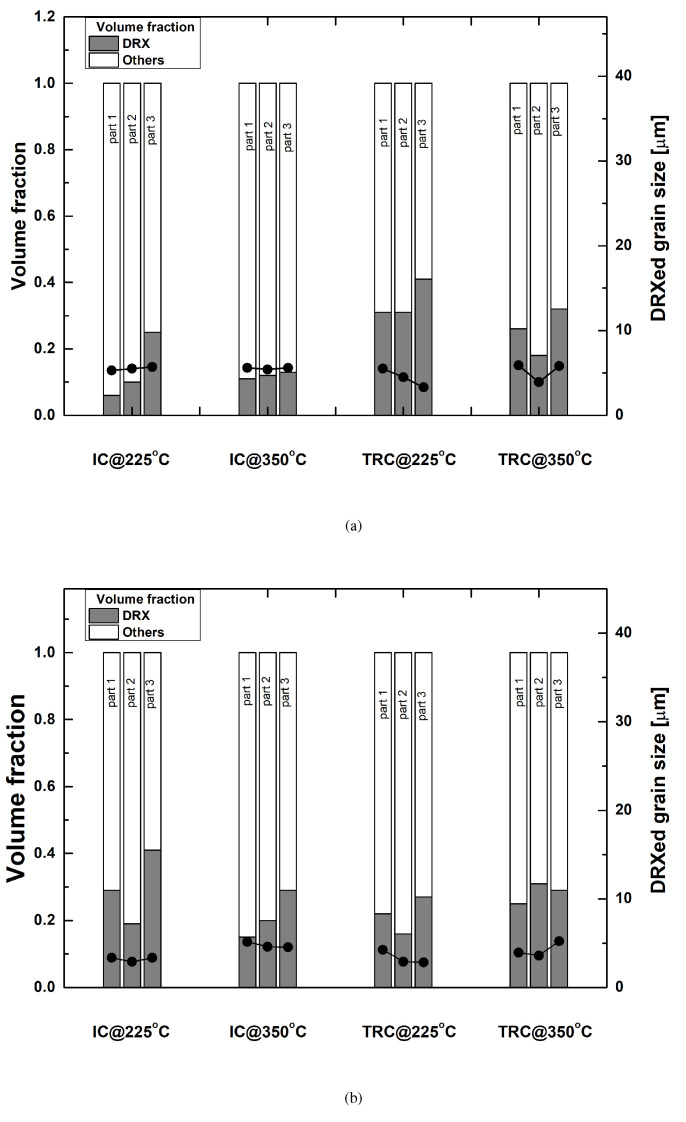
Volume fractions of small and large grains and their average grain size with each position of drawn cups, according to the deformation rates of (**a**) 30 mm/min, and (**b**) 50 mm/min.

Variations of the large and small grains reveal microstructural features of deformation and DRX. The criterion for recrystallized grains obtained from Figure 14 and small grains less than 300 pixels are used to detect DRX grains. Figure 15 summarize volume fraction of DRX grains and their grain size. Part 3 usually possessed a greater volume fraction of DRXed grains than Parts 1 and 2. This trend remained dominant as the deformation rate increased from 30 mm/min to 50 mm/min.

**Figure 16:**
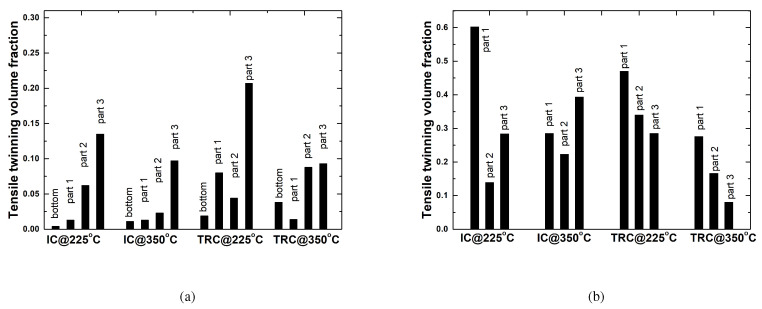
Volume fractions of tensile twin boundaries at each position of drawn cups, according to deformation rates of (**a**) 30 mm/min, and (**b**) 50 mm/min.

Tensile twins play an important role in the effort to understand deformation behaviors during deep drawing. The volume fractions of the tensile twin boundaries are summarized in Figure 16. Twinned fractions of the bottom regions are negligible. Increased numbers of tensile twins are more evident at a deformation rate of 50 mm/min when compared to the number at 30 mm/min. When considering the same deformation rate, a low working temperature of of 225 °C is favored for twining activation compared to a high temperature of of 350 °C. The Part 3 region possessed a greater number of tensile twins than Parts 1 and 2 at a deformation rate of 30 mm/min. This implies that thickening in the flange is the main sources of tensile twins. At a deformation rate of 50 mm/min, strong activation of tensile twins was observed in Part 1 with bending deformation. The volumes of tensile twins in Part 3 were smaller when compared to that in Part 1. This is associated with more activation of non-basal slips in Part 3 rather than twinning due to elevated temperature.

Large grains are usually favorable for twinning activation, compared to small grains. Grain size constraints on twin expansion in hexagonal close packed crystals was studied, and it was also found that twinning stress increases with decreasing grain size [44]. There are some variations in twinning volume fraction with cup position. During deep drawing of Mg-Al-Mn system at a drawing speed of 50 mm/min at 225 °C, it is evident that more twinning was observed in the IC sheets with large grains than in the TRC sheets with small grains. Under a decreased drawing speed of 30 mm/min at 225 °C, it is interesting to note that the Part 1 and Part 3 of the TRC sheets revealed more twinning activation, as compared to those of the IC sheets. When considering that there is some difference in distribution of AlMn dispersions between TRC and IC sheets, the refined dispersions seem to affect deformation mode of twinning, depending on deformation rate. In our previous studies of Mg–Al–Mn–Mm and Mg–Al–Mn–Ca systems [22,28], grains and AlMn particles of TRC sheets are smaller than those of IC sheets and this resulted in better yield and tensile strengthes in the TRC sheets. It is also evident that TRC sheets possess lower elongation than IC sheets, implying trade-off of strength and elongation. This is also found in the Mg–Al–Mn system, as shown in Figure 3. In other magnesium alloys with dispersion (Mg–Al–Zn–Ca systems), the addition of Ca increased volume fraction of dispersions and refined overall grain size [45]. With an increase in Ca addition, the yield strength increased and elongation decreased. Usually, dispersed particles prevented dislocation motion during deformation, and prohibited grain boundary motion during annealing. Various precipitates or dispersions affect the deformation modes of magnesium alloys [46]. A better understanding of the structure, morphology, and orientation of precipitates or dispersions is important for optimization of mechanical properties. Strength mechanism caused by interaction between twin growth and precipitates was also studied in detail [47]. Such studies on the interactions between second phases and deformation modes, including slips and twins, are not trivial. Detailed investigation on interactions of deformation modes with dispersions is left as further studies.

**Figure 17:**
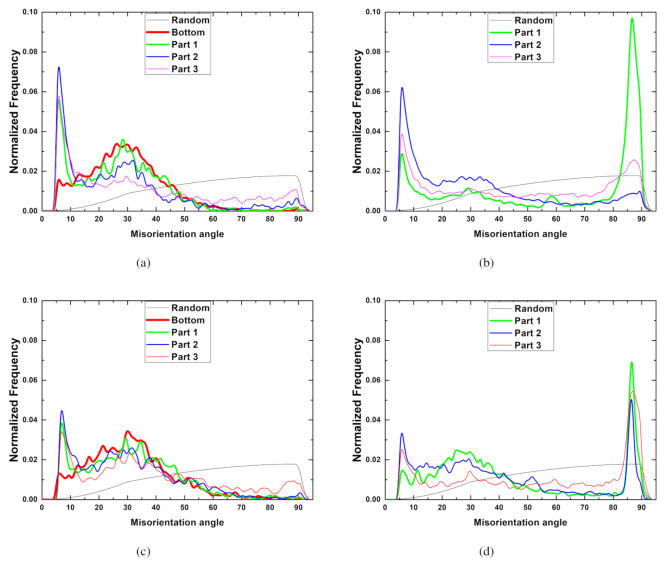
Frequency of misorientation distribution at various positions of drawn cups (IC sheets). Deformation rates and temperatures: (**a**) 30 mm/min, and (**b**) 50 mm/min at 225 °C, and (**c**) 30 mm/min, and (**d**) 50 mm/min at 350 °C

Macroscopic microstructural evolution can be understood using misorientation distribution. The typical normalized frequency of the misorientation distribution of IC sheets is shown in Figure 17. Overall, there are three distinct peaks in the misorientation distribution at a deformation rate of 30mm/min. The first peak between 5° and 10° comes from the subdivision due to plastic deformation. The second set of peaks around 30° is also found. Dominant double twin boundaries, $<10\bar{1}1>-<10\bar{1}2>$ are known as $38{}^{\circ}<11\bar{2}0>$ in magnesium alloys. Although minor double twin boundaries of $30.1{}^{\circ}<11\bar{2}0>$ are also reported [31], the nucleation of 30° <0001> grain boundaries seems to be more reasonable [48], when considering initial state at the bottom position. In this study, the frequencies of near 30° are twice greater than those of 38°. The third peak is observed near 86°, and this is associated with the tensile twins of $86{}^{\circ}<11\bar{2}0>$. A high frequency for tensile twins is mainly observed in Part 3, as discussed above. For a comparison, a random distribution is also included. At a deformation rate of 50 mm/min, peaks near 86° are most dominant, in addition to a high frequency at low angle misorientations, implying strong activation of tensile twins and grain subdivision, respectively. Part 1 is the main location of tensile twins at a deformation rate of 50 mm/min. It is also important to note the decrease in the peaks near 30° at a deformation rate of 50 mm/min (Figure 17a), compared to those at a deformation rate of 30 mm/min (Figure 17b). At a higher temperature of 350 °C, low angle frequency due to subdivision decreases. Overall, deformation rate is more critical to determine the misorientation distribution than temperature. At increased deformation temperature, low angle frequency decreases due to recovery of deformed structures. The trends of misorientation distributions of TRC sheets are similar to those of IC sheets, and they are not shown here.

## 5. Conclusions

The mechanical properties and textural and microstructural evolutions of wrought magnesium alloys of Mg-Al-Mn during deep drawing were investigated in detail using EBSD and TEM.

- Twin-roll casted (TRC) sheets had much finer precipitates and smaller grains than ingot-casted (IC) sheets. At room temperature, TRC sheets demonstrated greater strength and elongation than IC sheets. At elevated temperatures of 225 °C, and 350 °C, the IC sheets revealed better elongation than TRC sheets.
- During deep drawing, the bottom bent region, cup wall, and top flange experienced different deformation history, and dominant deformation modes varied with position. Most of the thinning of the drawn cups was observed near the bent, and most thickening near the top flange.
- The deformation rate is important to activate tensile twins both near the bent area and the flange. Tensile twins are much more evident at a deformation rate of 50 mm/min than at 30 mm/min. IC sheets possessed more tensile twins than TRC sheets.
- The working temperature dominantly affects deformation mechanisms and a refined grain structure caused by DRX during deep drawing. In particular, at a temperature of 225 °C, the refined DRXed grains in the flange is associated with twinning followed by DRX. At 350 °C, the DRXed grains in the flange reflected greater activation of non-basal slips rather than twins.

## Figures and Tables

**Table 1:** Deep drawing conditions.

	AM31
Punch diameter $\left[\mathrm{mm}\right]$	37
Specimen thickness $\left[\mathrm{mm}\right]$	0.6
Drawing speed $\left[\mathrm{mm}/\min\right]$	30, 40, 50
Temperature $\left[{}^{\circ}C\right]$	200∼350
Blank holding force $\left[\mathrm{kN}\right]$	5∼12
Blank diameter $\left[\mathrm{mm}\right]$	74
